# A predictive model for non-completion of an intensive specialist obesity service in a public hospital: a case-control study

**DOI:** 10.1186/s12913-019-4531-1

**Published:** 2019-10-24

**Authors:** Evan Atlantis, Fang Lin, Sulak Anandabaskaran, Paul Fahey, Nic Kormas

**Affiliations:** 1 School of Nursing and Midwifery, Western Sydney University, Penrith, New South Wales Australia; 2 School of Medicine, The University of Adelaide, Adelaide, South Australia Australia; 3 Campbelltown and Camden Hospitals, Campbelltown, New South Wales Australia; 4 Concord Repatriation General Hospital, Concord, New South Wales Australia; 5 School of Science and Health, Western Sydney University, Penrith, New South Wales Australia

**Keywords:** Dropout, Attrition, Weight management, Severe obesity, Weight-loss, Multidisciplinary

## Abstract

**Background:**

Despite the growing evidence base supporting intensive lifestyle and medical treatments for severe obesity, patient engagement in specialist obesity services is difficult to achieve and poorly understood. To address this knowledge gap, we aimed to develop a model for predicting non-completion of a specialist multidisciplinary service for clinically severe obesity, termed the Metabolic Rehabilitation Programme (MRP).

**Method:**

Using a case-control study design in a public hospital setting, we extracted data from medical records for all eligible patients with a body mass index (BMI) of ≥35 kg/m^2^ with either type 2 diabetes or fatty liver disease referred to the MRP from 2010 through 2015. Non-completion status (case definition) was coded for patients whom started but dropped-out of the MRP within 12 months. Using multivariable logistic regression, we tested the following baseline predictors hypothesised in previous research: age, gender, BMI, waist circumference, residential distance from the clinic, blood pressure, obstructive sleep apnoea (OSA), current continuous positive airway pressure (CPAP) therapy, current depression/anxiety, diabetes status, and medications. We used receiver operating characteristics and area under the curve to test the performance of models.

**Results:**

Out of the 219 eligible patient records, 78 (35.6%) non-completion cases were identified. Significant differences between non-completers versus completers were: age (47.1 versus 54.5 years, *p* < 0.001); residential distance from the clinic (21.8 versus 17.1 km, *p* = 0.018); obstructive sleep apnoea (OSA) (42.9% versus 56.7%, *p* = 0.050) and CPAP therapy (11.7% versus 28.4%, *p* = 0.005). The probability of non-completion could be independently associated with age, residential distance, and either OSA or CPAP. There was no statistically significant difference in performance between the alternate models (69.5% versus 66.4%, *p* = 0.57).

**Conclusions:**

Non-completion of intensive specialist obesity management services is most common among younger patients, with fewer complex care needs, and those living further away from the clinic. Clinicians should be aware of these potential risk factors for dropping out early when managing outpatients with severe obesity, whereas policy makers might consider strategies for increasing access to specialist obesity management services.

## Background

Global trends in the prevalence of severe obesity present an enormous clinical burden, challenging health care systems in high-income countries [1, 2]. In Australia, the prevalence of severe obesity defined using a body mass index (BMI) of ≥35 kg/m^2^ has likely tripled since 1980 [3]. Consequently, the total direct cost (health care and non-health care) of overweight and obesity to the Australian economy in 2005 was estimated at $21 billion annually [4]. The proportion of the average annual health expenditure is probably 50% higher for people with severe obesity compared to people with a healthy BMI of 18.5 to 24.9 kg/m^2^ [5]. Effective obesity management services for people living with from severe obesity and its complications could return significant health and economic benefits.

The complexity of severe obesity is challenging to manage in the primary care setting alone, especially in the presence of multiple medical, psychological, and physical comorbidities [6, 7]. These complex health care needs may be more appropriately managed in specialist obesity services [7–9]. Specialist obesity services (or ‘clinics’) typically provide physician-led multidisciplinary team (MDT) care utilising intensive lifestyle interventions and psychological support, as well as varying levels of access to weight-loss pharmacotherapies and bariatric surgery [10].

There is a growing evidence base supporting the effectiveness of a specialist obesity services including non-surgical MDT care, weight-loss pharmacotherapies, and bariatric surgery for improving a range of health outcomes in patients with clinically severe obesity [11–14]. Patients with severe obesity lose approximately 6% of their initial body weight after 12 months of non-surgical specialist obesity services [11, 12]. Conversely, non-completion rates of 30–60% over this period weakens this evidence base [15–20]. Studies have identified a number of possible predictors of non-completion such as younger age, socio-economic disadvantaged, and less medical complications [15–18, 20–22], as well as depression or anxiety [16, 18, 22]. Residential distance to services is likely to be another important predictor of non-completion of specialist obesity care programmes, given that musculoskeletal disorders are highly prevalent in patients with clinically severe obesity [23]. Given the substantial differences between these treatment programmes and settings, the applicability of this evidence in the Australian health system is unclear. Therefore, we investigated potential predictors of non-completion in a well-established specialist obesity service in an Australian public hospital setting. Potential predictors investigated include an extensive range of routine data collections on demographic information, residential distance from the clinic, anthropometry, smoking status, medical history, medically diagnosed conditions, medication use, and glycaemic control.

## Methods

We present this paper according to the STROBE guidelines for reporting observational studies [24] and the Journal’s formatting requirements. We previously presented the findings of this research at the ANZOS-OSSANZ-AOCO Joint Annual Scientific Meeting 2017 [25].

### Study design, setting, and participants

Using a case-control study design in a public hospital setting, we aimed to develop a predictive model for non-completion of a clinical obesity service, termed the Metabolic Rehabilitation Programme (MRP). The MRP is a specialist obesity service in an outpatient public hospital setting in the South Western Sydney Local Health District, which covers some of the most socio-economically disadvantaged communities in the state of New South Wales, Australia. Patients were referred to the MRP by medical practitioners, including general practitioners, specialists in hospitals, and specialists in primary care.

We extracted data from medical records for all of the eligible patients referred to the MRP between 2010 and 2015. To be considered to enter the service during that period, patients had to be: aged 18 years or older; have a BMI of at least 35 kg/m^2^ with either type 2 diabetes and/or fatty liver disease; and be committed to engage in the treatment programme and attend regular monthly clinic appointments. Exclusion criteria were: pregnancy; conditions associated with unintentional weight loss such as malignancy; and home oxygen therapy. Though patients could have engaged in the MRP for up to 24 months, a minimum treatment period of 12 months was recommended.

### Treatment programme

The MRP is an integrated physician-led MDT model of care for managing patients with complex and severe obesity [11, 26]. It is consistent with the model of care recommended in our recently published expert consensus, but is unique in the Australian health system in terms of staff resources and onsite exercise supervision [10]. Depending on the predominant complication, patients were enrolled either into the diabetes MRP or the fatty liver MRP. Both of the MRP clinics included physician, diabetes educator (for the diabetes MRP only), dietitian, clinical psychologist, and exercise physiologist/physiotherapist staff resources. The two programmes were available to patients for up to 24 months, after which they were typically discharged and referred back to their primary care doctor. Approximately 10% of suitable patients were referred for publicly funded bariatric surgery after 12 months of MRP. Due to the high rate of significant sleep disordered breathing discovered in the early years of this programme, a subsidised referral pathway was established with a local sleep physician to provide assessment and management of a clinically significant sleep disorder such as OSA or obesity hypoventilation syndrome. A detailed description of the key features of the MRP model of care has recently been published [27].

### Baseline data collections

We hand searched medical records of eligible patients for data extraction. Informed by predictors hypothesised in previous research [15–18, 20–22], we extracted the following routine baseline data collections for analysis: demographic information (age, gender, and residential distance from the clinic); anthropometry (weight, height, and waist circumference); and medical status including blood pressure, number of complications, OSA, use of current continuous positive airway pressure (CPAP) therapy, and current use of medications. Non-alcoholic fatty liver disease was assessed and monitored by the hepatologist. To determine the presence of depression and clinically severe anxiety, we used present medical history and current use of antidepressants. We categorised smoking status data into ‘current smoker’, ‘previous smoker’, or ‘non-smoker’ groups.

### Outcome data

We defined completion as patients who started and continued with the MRP for at least 12 months. As described above, a minimum treatment period of 12 months was recommended. This duration was chosen based on evidence from our previous research work in a similar MRP delivered in the Sydney Local Health District suggesting that improvements in anthropometric (e.g. weight-loss of 6–8%) and metabolic outcomes maximises at approximately 12 months [11, 26]. We also considered findings of a recent systematic review suggesting that at least 12 months of lifestyle intervention is required to achieve clinically significant weight loss of 5–10% in patients with severe obesity [28]. Non-completion status (case definition) was coded for patients who started but left within 12 months of the MRP. The primary outcome, non-completion of 12 months of MRP, was coded as a binary variable.

### Statistical analysis

Baseline characteristics are described using counts and percentages for categorical variables, means and standard deviations for numeric variables, with comparisons between groups using chi-square tests and independent samples t-tests, respectively. We describe *p*-values less than 0.05 as statistically significant and all p-values less than 0.25 as potentially predictive [29]. To help identify a set of key independent predictors of non-completion, we fitted a multivariable logistic model predicting non-completion using all predictors of interest. We started by including four of the statistically significant predictors (age, residential distance, OSA and CPAP), diabetes status, insulin medication and clinically severe anxiety, which were potentially predictive (*p* < 0.25) of association. We further included depression, as we hypothesised this could also impact on non-completion [16, 18, 22]. We excluded number of years since diagnosis of diabetes, even though its *p*-value was < 0.25, as it was not applicable to one third of the sample. To resolve the potential clinical overlap between OSA and CPAP, we report two final models: Model 1 including CPAP treatment of OSA (49/113 cases); and Model 2 including the diagnosis OSA. Missing data were noted and then excluded from the analysis. The main analyses were conducted using SPSS software. The relative predictive power of the fitted models was compared based on their received operating characteristic (ROC) curves using an appropriate online calculator (http://vassarstats.net/roc_comp.html).

## Results

### Baseline characteristics

Between 2010 to 2015, 239 patients were enrolled in the MRP. After excluding duplicate records (*n* = 4), baseline BMI < 35 kg/m^2^ (*n* = 9), previous bariatric surgery (*n* = 3), death (*n* = 1), and inability to commit to the MRP determined on initial consultation (n = 3), the final number of eligible data records for analysis was for 219 participants. Within this group, 56% had entered the diabetes MRP and 44% had entered the fatty liver MRP. Seventy-eight patients (35.6%) were identified as non-completers. Baseline characteristics of the MRP participants are presented both for all participants and for the 12-month completers and non-completers separately (Table 1).

**Table 1:** Baseline characteristics of patient enrolled in the MPR and comparison of predictors of completer and non-completers

Patients	All (*N* = 219)	Non- Completers (*n* = 78)	Completers (*n* = 141)	*P*-value
Age, years	52 (14)	47 (15)	55 (13)	**< 0.001**
Gender, male	96 (44%)	37 (47%)	59 (42%)	0.42
Distance from clinic, km	19 (14)	22 (14)	17 (14)	**0.018**
Weight, kg	140 (38)	143 (40)	139 (37)	0.48
BMI, kg/m^2^	50 (11)	50 (12)	49 (11)	0.58
Obese class II (BMI ≥35.00 to 39.99)	37 (17%)	12 (15%)	25 (18%)	0.66
Obese class III (BMI ≥40.00)	182 (83%)	66 (85%)	116 (82%)	
Waist circumference, cm (*n* = 202)	136 (19)	137 (21)	136 (18)	0.79
Smoking status				0.86
Never smoked	142 (65%)	50 (64%)	90 (65%)	
Current smoker	22 (10%)	7 (9%)	15 (11%)	
Previous smoker	55 (25%)	21 (27%)	34 (24%)	
Systolic blood pressure, mmHg	128 (16)	127 (16)	129 (16)	0.42
Diastolic blood pressure, mmHg	76 (13)	76 (11)	76 (14)	0.86
Depression	57 (26%)	22 (28%)	35 (25%)	0.59
Anxiety	9 (4.1%)	1 (1.3%)	8 (5.7%)	0.16
OSA (*n* = 218)	113 (52%)	33 (42%)	80 (57%)	**0.050**
CPAP (*n* = 218)	49 (23%)	9 (12%)	40 (28%)	**0.005**
Diabetes (*n* = 218)	141 (65%)	44 (56%)	97 (69%)	0.057
No. of years since diagnosis of diabetes (*n* = 140)	7.8 (7.2)	6.8 (7.1)	8.3 (7.2)	0.24
Medications				
Insulin	48 (22%)	13 (17%)	35 (25%)	0.16
Metformin	121 (55%)	43 (55%)	78 (55%)	0.98
No. of antihypertensive				0.68
0	66 (30.1%)	24 (30.8%)	42 (29.8%)	
1	59 (26.9%)	25 (32.1%)	34 (24.1%)	
2	50 (22.8%)	15 (19.2%)	35 (24.8%)	
3	30 (13.7%)	9 (11.5%)	21 (14.9%)	
≥ 4	14 (6.4%)	5 (6.4%)	9 (6.4%)	
Blood biochemistry				
HbA1c, % (*n* = 174)	7.1 (2.4)	7.3 (2.4)	7.1 (2.4)	0.57
HbA1c, mmol/L (*n* = 174)	78 (26)	80 (26)	77 (26)	0.57

Data are mean (standard deviation) unless otherwise specified; *BMI* body mass index; HbA1c, glycosylated haemoglobin; *p*-value for independent samples t-test for continuous variables or for chi-square test for categorical variables

### Predictors of non-completion

As shown in Table 1, we found younger age, greater residential distance from the clinic, and less complications (OSA and CPAP) showed statistically significant evidence of association with non-completion. We also detected weak evidence of an association between diabetes and non-completion. There were no significant differences found in the other characteristics we explored.

### Independence of the predictors

The probability of non-completion could have been independently associated with age, residential distance, and either OSA or CPAP (Table 2). Our multivariate analyse revealed that diabetes status (*p* = 0.893; OR 0.95, 95% CI 0.48–1.91) and then insulin (*p* = 0.380; OR 0.71, 95% CI 0.33–1.52) added little if any independent information on non-completion and were removed from further consideration. Specifically, we found diabetes status was strongly associated with age in this population; every additional year of age had a 5% higher odds of diabetes diagnosis than the previous age (*p* < 0.001; OR 1.05, 95% CI 1.03–1.08). We next removed depression (*p* = 0.576; OR 1.22, 95%CI 0.61–2.46) and then anxiety (*p* = 0.265; OR 0.29, 95% CI 0.03–2.58) from the multivariable model because of little statistical evidence of association and wide uncertainty in effect sizes (odds ratios). These final fitted models are shown in Table 2. We have retained residential distance in these models despite *p*-values greater than 0.05. The relatively small p-values and tight confidence intervals on the odds ratio suggest it is plausible that residential distance offered some independent information about completion.

**Table 2:** Fitted models for predicting 12-month non-completion of the MRP based on patients’ baseline characteristics

Predictor	Model 1		Model 2	
	OR (95% CI)	*p*-value	OR (95% CI)	*p*-value
Age, years	0.96 (0.94–0.99)	0.001	0.97 (0.94–0.99)	0.001
Distance from clinic, km	1.02 (1.00–1.04)	0.065	1.02 (1.00–1.04)	0.084
CPAP	0.30 (0.13–0.68)	0.004		
OSA			0.56 (0.31–1.00)	0.051

*CPAP* continuous positive airway pressure, *OSA* obstructive sleep apnoea

**Fig. 1:**
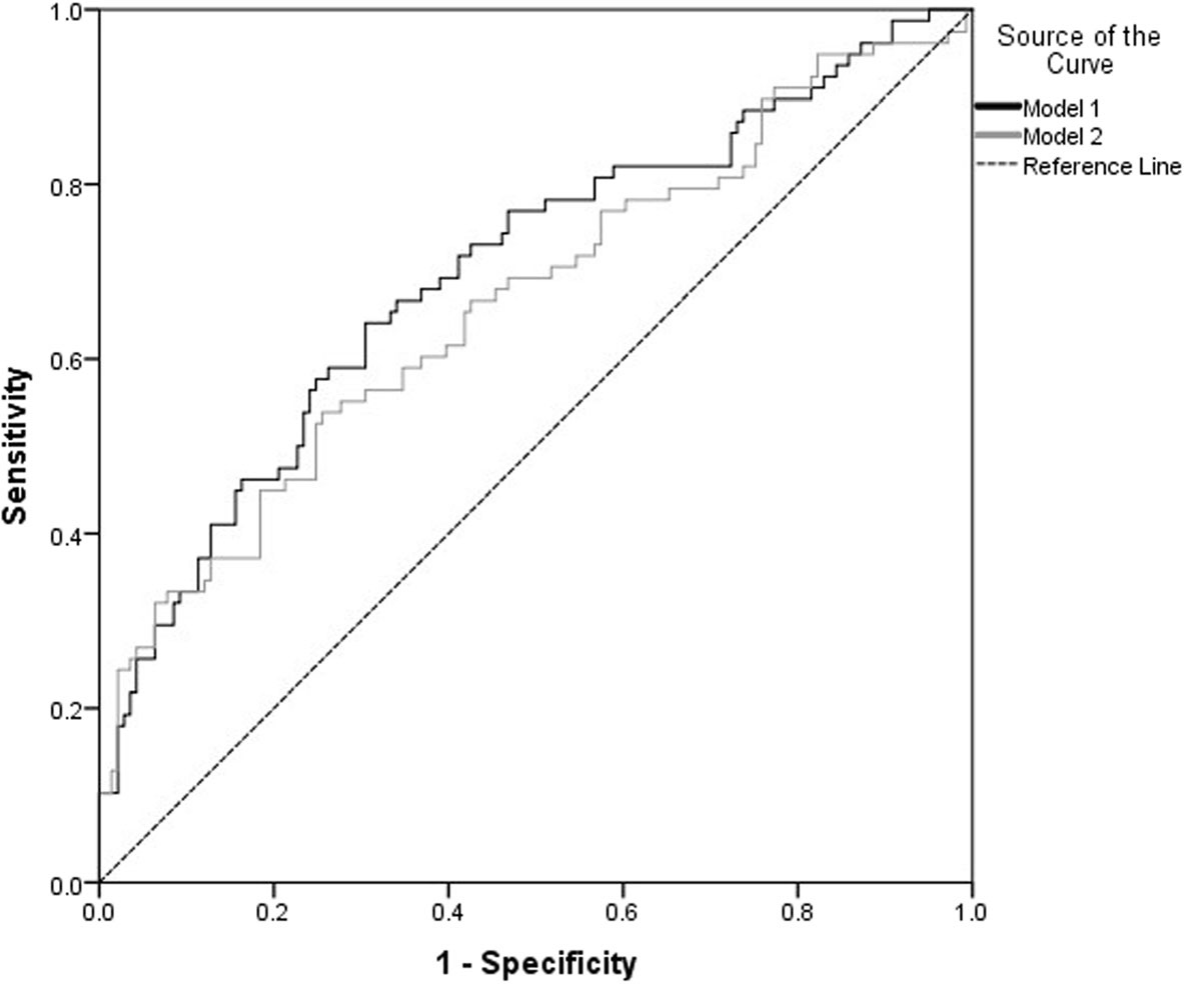
ROC area under curves for fitted models (Model 1 versus Model 2)

Our results show that once you take into account age, residential distance, and OSA or CPAP then none of the other variables add any further information about likelihood of non-completion. The probability of non-completion was inversely associated with age (*p* = 0.001), residential distance from the clinic (*p* = 0.065), and CPAP (*p* = 0.004) in Model 1; and inversely associated with age (*p* = 0.001), residential distance from the clinic (*p* = 0.084), and OSA (*p* = 0.051) in Model 2. There was no statistically significant difference in the predictive power of the two models (ROC area under curves of 69.5% versus 66.4%, *p* = 0.57, Fig. 1).

## Discussion

This paper presents the results of the first study of potential predictors of 12-month non-completion of an intensive lifestyle (with supervised exercise intervention) and medical obesity management service for patients with severe obesity. Overall, we observed a non-completion rate of 36%. This is approximately 20% lower than previous studies in comparable real-world settings over 6 and 12 month periods [15, 20, 21]. This finding is clinically useful information given that our patients typically have extremely severe obesity (mean BMI of 50; 83% with a BMI ≥40, Table 1) limiting their physical capacity to travel to the clinic and participate in a comprehensive and intensive care plan which includes onsite supervised exercise sessions 2–3 times weekly. Consistent with previous research work conducted in other countries and settings [15, 16, 20, 21], we confirmed that younger aged patents with less medical complications are at increased risk of dropping out of specialist obesity services early. Despite having severe obesity, the health needs of these younger and less complex patients might be more appropriately addressed in primary care rather than in specialist obesity management clinics, especially where access to intensive services and treatments is severely limited [10]. We also found evidence that residential distance was positively associated with non-completion, which is broadly consistent with limited research on this issue from studies in the US [30] and UK [31]. There is consensus among the Australian physicians that geographical location is a major obstacle for most patients because regular travel to the few specialist obesity clinics located in major cities is considered prohibitive [10].

Unlike some previous studies [16, 18, 22], we found no statistical evidence that depression or anxiety predicts dropping out of specialist obesity services early. The MRP had included comprehensive support from a clinical psychologist at the time, which might have helped some patients with severe obesity and depression or anxiety engage in the intensive specialist services and treatments [22]. Future research is needed to confirm if comprehensive psychological support is important not only for addressing the mental health issues and needs of the patient but also for enhancing patient engagement in these services and treatments [32]. Despite no comprehensive evidence base on this issue, a range of experts agree that MDT care should include psychologists for comprehensive service delivery [7–10]. Furthermore, our observation that the prevalence of OSA and current CPAP were positively associated with completion of the MRP is consistent with at least one other study [20]. As with psychological support, the MRP patients with OSA had access to a specialist sleep physician in the primary health setting, which could have helped retain these patients. Similarly, the provision of on-site supervised exercise in the MRP could have also partially contributed to improvements in engagement and mood among patients with depression or anxiety. Regular exercise programmes are believed to improve mood and increase physical activity level in people with mild-to-moderate depression [33].

The results from our study have direct implications for clinical practice. Whilst the MRP has been shown to be effective in the management of severe obesity in Australia [11, 26], it is important to optimise the engagement of these patients to improve their outcomes but also for the efficacy of such services. Therefore, screening for predictors of non-completion could be useful in identifying risk factors that could be targeted with effective prevention strategies. Existing and future obesity management clinics should consider the potential adverse impact of the availability of specialist treatments and services on patient engagement. There are enormous differences in terms of patient access to, and composition of, specialist obesity management services and treatments in Australia and other countries [10, 12]. Our findings suggest that policy makers should consider developing better access to existing clinics by improving transportation options or perhaps exploring novel telehealth delivery of some of the treatments [34], especially for rural and socio-economically disadvantaged areas where specialist obesity management services are absent [10].

Although this paper presents clinically important new findings in a real-world hospital setting, they should be interpreted with caution in consideration of several study limitations and potential risks of bias. For instance, our use of a case-control study design likely resulted in a unique sample of motivated patients not representative of the target population. The MRP entry criteria at the time was very strict and more selective than most of the other specialist obesity services nationwide [10]. Our patients were likely highly motivated because they were aware of the expected commitment to the high intensity services and treatments in the MRP. Few patients may have also been motivated because of the possibility of accessing publicly funded bariatric surgery after 12 months in the MRP. Although we selected a treatment period of 12 months for our analysis of predictors based on maximized weight-loss and metabolic outcomes from our previous research [11, 26], a systematic review concluded that weight-loss following several different lifestyle and pharmacotherapies plateaus at approximately 6 months, which limits the generalizability of our findings [35]. Our reliance on medical records for data extractions could have resulted in bias due to limited, incomplete, and non-standardised information from routine baseline assessments. Consequently, we were unable to explore a comprehensive range of potentially important reasons for non-completion. In particular, early weight-loss response (within the first 2 months of treatment) has been shown to predict weight-loss maintenance at 6 and 12 months [36, 37]; completion of a dietary-based specialist obesity service [38]; and engagement in post bariatric surgery care [30]. Future research is needed to better understand the depth and breadth of reasons for non-completion in specialist obesity services.

## Conclusions

In conclusion, our results suggest that non-completion of intensive specialist obesity services such as the MRP is most common among younger patients, those with fewer complex care needs, and those living further away from the clinic. Clinicians should be aware of these potential risk factors for dropping out of specialist obesity management services early when managing outpatients with severe obesity, whereas policy makers might consider strategies for increasing access to specialist obesity management services by improving transportation options or perhaps exploring novel telehealth delivery of some of the treatments, especially for areas where such services do not exist.

## Data Availability

The datasets generated and/or analysed during the current study are not publicly available due to their sensitive nature.

## Abbreviations

BMI: Body mass index
CI: Confidence interval
CPAP: Continuous positive airway pressure
MDT: Multidisciplinary team
MRP: Metabolic Rehabilitation Programme
OR: Odds ratio
OSA: Obstructive sleep apnoea
